# Assessing accuracy and artefacts in proton stopping power ratio images across four computed tomography imaging workflows using a head-sized electron density phantom

**DOI:** 10.1093/rpd/ncaf159

**Published:** 2026-03-13

**Authors:** Erik Pettersson, Anne Thilander Klang, Callum Gillies, Matthew Clarke, Anna Bäck

**Affiliations:** Department of Medical Radiation Sciences, Institute of Clinical Sciences, Sahlgrenska Academy, University of Gothenburg, SE-413 45 Gothenburg, Sweden; Therapeutic Radiation Physics, Department of Biomedical Engineering and Medical Physics, Sahlgrenska University Hospital, SE-413 45 Gothenburg, Sweden; Department of Medical Radiation Sciences, Institute of Clinical Sciences, Sahlgrenska Academy, University of Gothenburg, SE-413 45 Gothenburg, Sweden; Diagnostic Radiation Physics, Department of Biomedical Engineering and Medical Physics, Sahlgrenska University Hospital, SE-413 45 Gothenburg, Sweden; Proton Therapy Physics, University College London Hospitals NHS Foundation Trust, London, NW1 2BU, United Kingdom; Christie Medical Physics and Engineering, The Christie NHS Foundation Trust, Manchester, M20 4BX, United Kingdom; Department of Medical Radiation Sciences, Institute of Clinical Sciences, Sahlgrenska Academy, University of Gothenburg, SE-413 45 Gothenburg, Sweden; Therapeutic Radiation Physics, Department of Biomedical Engineering and Medical Physics, Sahlgrenska University Hospital, SE-413 45 Gothenburg, Sweden

## Abstract

The efficacy of proton beam therapy is limited by stopping power ratio (SPR) prediction uncertainties in patient tissues. This study compared image artefacts and SPR prediction accuracy across a single-energy computed tomography (SECT) and three dual-energy computed tomography (DECT) workflows: SECT with a clinical Hounsfield look-up table (HLUT), two commercial DECT algorithms (DirectSPR and MMSim), and an in-house developed model applied to material density (MD) images, called MD-SPR. SPR images of a head-sized phantom with 24 inserts of tissue surrogate and non-tissue materials were evaluated for image artefacts and compared with measured reference SPRs of the inserts. The root-mean-square SPR differences for tissue surrogates were 0.011 (HLUT), 0.005 (DirectSPR), 0.007 (MMSim), and 0.005 (MD-SPR). For non-tissue materials, the differences were 0.167, 0.028, 0.034, and 0.011, respectively. These results indicate that DECT-based SPR prediction workflows, particularly MD-SPR, can reduce both image artefacts and range uncertainties, compared with a SECT-based HLUT workflow.

## Introduction

The efficacy of proton beam therapy is constrained by uncertainties in the range of the proton beam within the patient [1]. During treatment planning, this range is estimated by integrating the stopping power ratio (SPR) along the beam path [2]. Reducing SPR uncertainties has been shown to decrease the volume receiving the prescribed dose, leading to clinically relevant dose reductions in organs at risk situated close to the target volumes [3], as well as increased estimated quality-adjusted life expectancy [4]. A major component of the range uncertainties arises when the SPRs of the patients’ tissues are predicted from single-energy computed tomography (SECT) images using heuristic, piecewise-linear Hounsfield look-up tables (HLUTs) [5, 6]. This is partly due to the lack of a one-to-one relationship between CT numbers (in Hounsfield units) and SPRs for human tissues [7]. Range uncertainties are further increased when the proton beam traverses ancillary devices made of non-tissue materials, such as polymers in implants and immobilisation equipment for radiotherapy [8, 9]. Moreover, the range of the proton beam is affected by artefacts in the CT images, such as dark streaks between dense bones. Although these artefacts are well known, the evaluation of the associated range uncertainties is non-trivial. This is due to the widely varying geometry of the patient and ancillary equipment, but also the fact that the artefacts can depend on the CT technique and scan settings used [10].

To decrease range uncertainties, SPR prediction based on multi-energy CT (MECT) has been extensively studied [11, 12]. MECT estimates the photon beam attenuation properties of a material by acquiring images at two or more energies, either by varying the tube voltage or using energy-discriminating detectors [13]. The term MECT includes the terms dual-energy CT (DECT) and spectral CT, using the nomenclature of AAPM Task Group 299 [14]. The estimated photon attenuation coefficient is often separated into two terms corresponding to the two main X-ray interaction processes in the diagnostic energy range (20–200 keV), i.e. Compton scattering and photoelectric absorption. This is often visualised in the MECT post-processing software as a pair of images, depending on the vendor and the specific MECT technique used. Some vendors provide relative electron density (RED) and effective atomic number (EAN) images, whereas others provide a pair of basis material density (MD) images, where the estimated photon attenuation coefficient is described as a linear combination of two equivalent densities of two basis materials. The choice of the two basis materials is arbitrary, but they should have sufficiently different photon attenuation characteristics while avoiding absorption edges in the energy spectra used for the scans. A common example of MDs used in clinical MECT is water and iodine. When this so-called basis material decomposition is performed in projection-space, prior to image reconstruction, streak artefacts caused by beam hardening can be reduced [13].

Many MECT-based SPR prediction workflows separate the SPR into two factors, the RED and the relative stopping number (RSN), as shown in Equation 1.


1 \begin{eqnarray*} SPR= RED\bullet RSN \end{eqnarray*}

This is an intuitive approach, as RED images often have been available directly from the MECT scanner or associated post-processing software. However, since there is no theoretical relationship between the RSN and EAN, the RSN has typically been obtained from EAN images using heuristic look-up tables [7, 15, 16]. This approach works for human reference tissues but not necessarily for non-tissue materials. Moreover, the use of EAN images comes with further drawbacks, firstly, as there are multiple definitions of the EAN [17, 18], and secondly, EAN images are susceptible to beam hardening artefacts [19, 20] that may propagate to the SPR images. Despite these disadvantages, studies have shown improved SPR prediction accuracy using RED and EAN, compared with SECT for both tissue surrogates and non-tissue materials [21, 22], as well as for animal tissue samples [23, 24]. At the time of writing, two commercially available DECT-based SPR prediction algorithms exist, DirectSPR (Siemens Healthineers, Erlangen, DE) and MMSim (Philips Healthcare, Best, NL). Comparisons between these methods are scarce in the literature.

An alternative approach is a DECT-based workflow using the MD-SPR model developed by Pettersson *et al.* [25], which is based on a heuristic 2D, non-parametric surface fitting that maps theoretical water and iodine MDs to SPR values.

Previous studies have primarily assessed SPR accuracy under idealised conditions, i.e. by evaluating different materials separately in a homogeneous phantom [21, 23, 24]. In addition to the performance of the SPR prediction algorithm itself, the SPR accuracy is dependent on the underlying image quality and the MECT technique used, e.g. DECT based on consecutive scans, dual-layer detectors (DLCT), or fast kilo-voltage switching (FKS-DECT). Furthermore, since patient scans often contain inhomogeneities including dense bone and image artefacts, it is important to evaluate the impacts of any such artefacts on SPR accuracy.

The purpose of this study was to compare SPR images generated using HLUT, DirectSPR, MMSim, and MD-SPR workflows under varying beam hardening conditions, with regard to streak artefacts and to quantify deviations between predicted SPR values and reference SPRs determined from proton beam measurements of both tissue surrogates and non-tissue materials.

## Materials and methods

### Phantom material inserts for stopping power ratio comparisons

This study included both tissue surrogates and non-tissue materials. Tissue surrogates were obtained from a Gammex AED phantom (Model 1467, Sun Nuclear – a Mirion company, Melbourne, FL, US), where the 2.85 cm diameter inserts were cut into 5 cm long cylinders. These materials, together with air (no insert) and liquid water, are hereafter referred to as ‘tissue surrogates’. Non-tissue material inserts were machined from polymers commonly used in applications such as implants, immobilisation devices, and phantoms used in both radiology and radiotherapy. A cylinder of bone cement was produced in-house by extrusion and compression into a mould, then machined into final dimensions. Due to limited availability of the bone cement, this insert measured 4.2 cm in length and 2.5 cm in diameter. All insert ends were smoothed with sandpaper. Further details of the materials are provided in the Results section.

### Reference stopping power ratio determination

The reference SPRs (${SPR}_{ref}$) of the material inserts were determined using a 220 MeV pencil beam from a clinical proton therapy system (ProBeam, Varian Medical Systems – a Siemens Healthineers Company, Palo Alto, CA, US) in combination with a Peakfinder detector system (PTW Dosimetry, Freiburg, DE). The Peakfinder system consists of two plane-parallel ionisation chambers separated by a flexible water-filled expansion chamber (bellows). One chamber remained stationary at the beam entrance, whereas the other was movable within the water-equivalent path length created by the bellows [26, 27]. Depth ionisation curves were measured with each material insert placed concentrically within the beam path. Measurements without any insert (${R}_{air}^{80}$) were performed at the beginning and end of each measurement session. The ${SPR}_{ref}$ was determined from the shift of the distal 80% level (${R}^{80}$) of the depth ionisation curves, as expressed in Equation 2:


2 $${\begin{eqnarray*} {SPR}_{ref}={k}_E\bullet \left(\frac{R_{air}^{80}+{L}_{insert}\bullet{SPR}_{air}^{220\ MeV}-{R}_{insert}^{80}\kern0.5em }{L_{insert}}\right). \end{eqnarray*}}$$

Equation 2 in this work is based on Equation 1 in Niepel *et al.* [24]. The length of each insert (${L}_{insert}$) was measured using a micrometer screw gauge (M320-50AA, Mitutoyo Corporation, Kanagawa, JP). The term ${L}_{insert}\bullet{SPR}_{air}^{220\ MeV}$ accounted for the air traversed by the proton beam when no insert was present, and was calculated for air and water at 20 °C and 101.3 kPa, using mass stopping powers from ICRU Report 90 [28].

An energy correction factor (${k}_E$) was applied to all materials with known elemental compositions (except for bone cement and CFR-PEEK, for which the elemental compositions were unknown). This factor accounts for the discrepancy between the beam energies used for the measurements (220 MeV) and SPR prediction (typically 100 MeV, see section “Computed Tomography”). A high energy is preferred for measurements to minimise the lateral size of the beam and the energy dependence within the inserts [6], whereas lower energies (e.g. 100 MeV) are often used in CT-based SPR prediction to minimise the range uncertainties caused by the energy dependence of the SPR across the entire beam path in the patient [29]. The correction factor, ${k}_E$, was defined as the ratio of the theoretically calculated SPRs at 100 and 220 MeV,


3 \begin{eqnarray*} {k}_E=\frac{SPR^{100 MeV}}{SPR^{220 MeV}}, \end{eqnarray*}

using the Bethe formula and ICRU-recommended elemental mean excitation energies (I-values) for use in compounds (ICRU Report 49) [30].

The uncertainty of the measured ${SPR}_{ref}$ was evaluated according to GUM principles using a Type B evaluation [31]. The main contributors were assumed to be the maximum permissible error (MPE) of the ionisation chamber position (MPE ±0.05 mm, according to Witt *et al.* [27]) and the measured length of the insert (MPE ±0.02 mm, according to the micrometre specifications). The MPEs were converted to standard uncertainties assuming triangular distributions [31]. The combined standard uncertainty was obtained by propagating these components using Equation 7 from Moyers *et al.* [32]. The expanded uncertainty was calculated with a coverage factor k = 2 (95% confidence). This evaluation assumes that the inserts are amorphous and free of large-scale inhomogeneities.

${SPR}_{ref}$ for air was calculated from tabulated values from ICRU Report 90 [28], and for liquid water was set to unity.

### Phantom configuration

**Figure 1:**
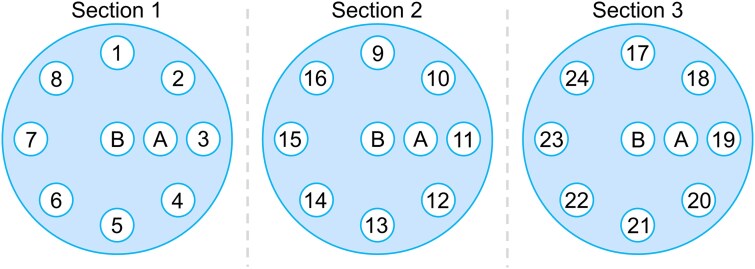
Illustration of the three transverse cross-sections of the 20 cm diameter electron density phantom used in this study. Three distinct sections of the phantom were formed by placing the 5 cm long inserts in clockwise descending order of SPRref, from PTFE in position 1 to air in position 24. The 16.5 cm long inserts of HE Brain (B) and HE General Adipose (A) extended through all three sections.

A single phantom was configured based on the 20 cm diameter, 16.5 cm long cylindrical head part of the AED phantom, with all 5 cm long inserts placed in the peripheral holes in clockwise descending order of ${SPR}_{ref}$, starting with PTFE at the 12 o’clock position. The eight peripheral insert positions in the phantom allowed for the inclusion of 24 materials in three separate sections with different beam hardening conditions (Fig. 1). Two 16.5 cm long tissue surrogate inserts (HE Brain and HE General Adipose) extended through the entire length of the phantom. These inserts were used for a focused quantitative evaluation of the influence of image artefacts under different beam hardening conditions.

### Computed tomography

The phantom was scanned with SECT and consecutive-scan DECT (Twin-spiral) using a SOMATOM go.Open Pro (Siemens Healthineers, Forchheim, DE). DLCT scans were performed with a Spectral CT 7500 RT (Philips Healthcare, Best, NL), and FKS-DECT scans were performed on a Revolution Apex (GE Healthcare, Waukesha, WI, US), for which the scan and reconstruction parameters were selected to match those of the clinical SECT scans (Table 1). All scanners were equipped with flat tabletops, and all scans were repeated three times for each imaging technique.

**Table 1:** Scan and reconstruction parameters for the image acquisitions.

CT Scanner	SOMATOM go.Open Pro		Spectral CT 7500 RT	Revolution Apex
Scan mode	SECT	Twin-spiral DECT	DLCT	FKS-DECT
Software version	syngo CT VA40A	syngo CT VA40A	5.0.1	cadence_ct_25.44
Tube voltage (kV)	120	Sn140 & 80	120	140 / 80
Bowtie filter	W1	W1	UB	Large Body
Total collimation width (mm)	38.4	38.4	40	40
Helical pitch	0.55	0.45	0.579	0.984
Rotation time (s)	1.0	1.0	0.5	0.8
Scan field-of-view (mm)	600	600	500	500
Display field-of-view (mm)	400	400	400	400
Image slice thickness (mm)	1.50	1.50	2.0	1.25
Convolution kernel	Qr40, iBHC(bone)	Qr40, iBHC(bone)	UB	Standard
Iterative noise reduction	SAFIRE, level 2	SAFIRE, level 2	Spectral level 5	ASiR-V, 60%
X-ray tube current (mA)	190	248 & 268	397–459	445
CTDI_vol,16 cm_ (mGy)	48.8	48.6	51.4–59.4	45.4^*^

SECT = single-energy CT, DECT = dual-energy CT, DLCT = dual-layer detector CT, FKS-DECT = fast-kV switching DECT, ASiR-V = adaptive statistical iterative reconstruction V, SAFIRE = sinogram affirmed iterative reconstruction. CTDI_vol,16cm_ = volume CT Dose Index with the ⌀16 cm PMMA phantom.

^*^The CTDI_vol,16cm_ was obtained by multiplying the CTDI_vol,32cm_ with 2.3741, according to Table 34 in the technical reference manual (5848882-1EN).

The SECT images from the SOMATOM go.Open Pro were converted into SPR images using a clinical HLUT obtained from a stoichiometric calibration for a proton beam energy of 100 MeV [33]. For this work, this conversion was performed using MATLAB (MathWorks, Natick, MA, US). The high energy (Sn140 kV) and low energy (80 kV) images from consecutive scans using the same scanner were imported into the post-processing software (syngo.via, version VB60B, Siemens Healthineers, Forchheim, DE), where automatic non-rigid registration was performed prior to generating RED and EAN images [21]. SPR images were then created using the DirectSPR application, which includes a patient-size correction as part of the image processing pipeline before the SPR calculation [34].

For the DLCT scans, the RED and EAN images were created based on the projection raw data on the CT console, which were then imported into the post-processing software (Multi-Modality Simulation workspace, MMSim, version 18.0.5) to create SPR images [35]. The EAN images were converted to RSN images using the ‘SPR_Bethe_Bourque’ setting, corresponding to Bourque *et al.* [15] (as recommended by Philips clinical science representatives), and the ‘Proton High Energy’ setting, corresponding to a beam energy of 200 MeV. The water and iodine MD images from the FKS-DECT scans were reconstructed as GSI data files, from which SPR images were created in MATLAB using the MD-SPR model from Pettersson *et al.* [25], with the following modifications:

- The mean excitation energies (I-values) for chemical elements in compounds and water from Bär *et al.* [36] were replaced with I-values from ICRU reports 49 [30] and 90 [28], respectively.
- The tissue compositions from the ICRP adult and paediatric voxel phantoms (ICRP Reports 110 and 143) [37, 38] were replaced with those from the newer mesh-type phantoms (ICRP Reports 145 and 156) [39, 40].
- The span of the locally weighted scatterplot smoothing (LOWESS) was increased from 15% to 25%.

**Figure 2:**
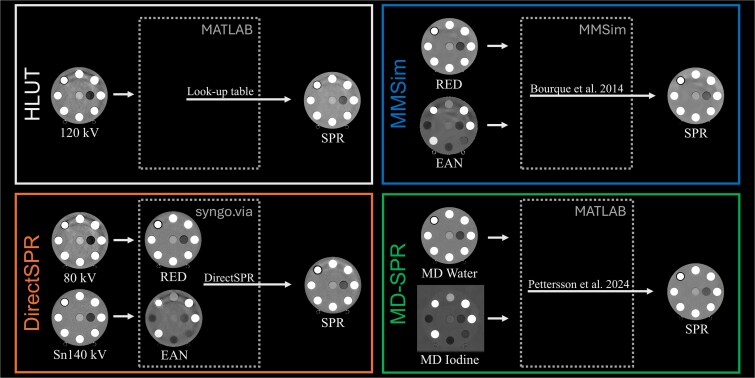
Visualisation of the four workflows used to generate SPR images using four different CT techniques. All images shown depict Section 1 of the 20 cm diameter electron density phantom. The polychromatic CT images (120 kV, 80 kV, and Sn140 kV) are displayed using a greyscale window of −100 to 100 HU, whereas the RED, EAN, and SPR images are displayed with grayscale windows spanning 0.9–1.1, 5–11, and 0.9–1.1, respectively. The water and iodine MD images are displayed using windows spanning from 900 to 1100 and −3 to 7 mg/cm^3^, respectively.

A visualisation of the four different workflows is shown in Fig. 2.

All SPR images were imported into a clinical treatment planning system, Eclipse (version 18.0, Varian Medical Systems, a Siemens Healthineers Company, Palo Alto, CA, US). A qualitative visual inspection of image artefacts (dark or light streaks in the homogeneous water-like material between the phantom inserts) was performed. Additionally, a quantitative analysis was conducted by evaluating the range (i.e. maximum ‘minus’ minimum) of the predicted SPR values for the HE General Adipose and HE Brain inserts (based on the mean VOI values) across the three sections in the phantom.

The mean SPRs were extracted from 3 cm long, 1.8 cm diameter cylindrical volumes of interest (VOIs) within each insert and averaged across the three repeated scans. The pooled standard deviation in each VOI across the three repeated scans were also extracted. The difference between the predicted SPR values and ${SPR}_{ref}$ was evaluated. Root-mean-square errors (RMSEs) were calculated separately for the tissue surrogates and non-tissue materials. Separate RMSEs were also calculated for the bone and soft tissue surrogates. Note that the 16.5 cm long HE General Adipose and HE Brain inserts were excluded from comparisons with ${SPR}_{ref}$ as these were too long for the proton beam measurements.

## Results

The expanded uncertainty of the measured ${SPR}_{ref}$ (calculated with a coverage factor k = 2, i.e. 95% confidence) ranged from ±0.0017 for Lung 300 to ±0.0025 for bone cement.

The largest energy correction factor (${k}_E$ = 1.003) was calculated for the PMP, PP, and PE polymers (all assumed to have the same elemental composition), while the smallest values (0.995 and 0.996) were found for PTFE and HE Cortical Bone, respectively (Table 2).

**Table 2:** Specifications and abbreviations for the phantom insert materials used in SPR comparisons.

							$\boldsymbol{SPR}-{\boldsymbol{SPR}}_{\boldsymbol{ref}}$			
Phantom section	Position	Insert material	Trade name, vendor	Material group	${\boldsymbol{k}}_{\boldsymbol{E}}$	${\boldsymbol{SPR}}_{\boldsymbol{ref}}$	HLUT	DirectSPR	MMSim	MD-SPR
	1	Polytetrafluoroethylene (PTFE)	PTFE G400, Guarniflon S.p.A., Castelli Calepio, IT	Non-tissue	0.995	1.779	−0.330	0.085	0.092	0.034
	2	Cortical bone	1450 HE Cortical Bone, Sun Nuclear, Melbourne, US	Tissue (bone)	0.996	1.695	0.015	−0.001	0.007	0.000
	3	CB2 + 50% CaCO_3_	480 CB2 + 50% CaCO_3_, Sun Nuclear	Tissue (bone)	0.997	1.430	0.002	−0.002	0.011	−0.004
1	4	Polyoxymethylene (POM)	TECAFORM AD natural (POM-H; Delrin®), Ensinger	Non-tissue	1.000	1.371	−0.186	0.024	0.020	0.005
	5	Carbon-fibre-reinforced PEEK (CFR-PEEK)	TECAPEEK MT CF30 (30%_vol_ carbon fibre), Ensinger	Non-tissue	–	1.300	−0.156	0.034	0.035	0.006
	6	CB2 + 30% CaCO_3_	484 CB2 + 30% CaCO_3_, Sun Nuclear	Tissue (bone)	0.999	1.266	−0.019	0.001	0.009	−0.003
	7	Polyetheretherketone (PEEK)	TECAPEEK natural, Ensinger GmbH, Nufringen, DE	Non-tissue	1.001	1.230	−0.111	0.021	0.034	0.004
	8	Bone cement	Palacos R + G pro, Heraeus Medical, Wehrheim, DE	Non-tissue	–	1.207	0.393	−0.004	0.039	0.009
	9	Polysulfone (PSU)	TECASON S natural, Ensinger	Non-tissue	0.999	1.167	−0.046	0.001	0.009	0.012
	10	Polymethyl methacrylate (PMMA)	Acrylic (Extruded), Ensinger	Non-tissue	1.001	1.163	−0.072	0.011	0.021	0.007
	11	Inner bone	1456 HE Inner Bone, Sun Nuclear	Tissue (bone)	0.999	1.152	0.017	0.006	0.014	0.002
2	12	Polyamide type 6 (Nylon)	TECAMID 6 natural, Ensinger	Non-tissue	1.002	1.146	−0.076	0.005	0.016	−0.005
	13	Polycarbonate (PC)	TECANAT natural, Ensinger	Non-tissue	1.001	1.144	−0.064	0.016	0.024	0.010
	14	Liver	1482 HE Liver, Sun Nuclear	Tissue (soft)	1.001	1.064	−0.009	−0.010	−0.002	0.000
	15	Polystyrene (PS)	Rexolite 1422, C-Lec Plastics, Philadelphia, US	Non-tissue	1.002	1.036	−0.039	0.010	0.026	0.002
	16	CT Solid Water	1451 HE CT Solid Water®, Sun Nuclear	Tissue (soft)	1.001	1.003	0.008	−0.007	0.001	−0.004
	17	Liquid water		Tissue (soft)	–	1.000	0.014	0.003	0.011	0.006
	18	Polyethylene (PE)	PE 1000 natural, Simona AG, Kirn, DE	Non-tissue	1.003	0.992	−0.036	−0.006	0.014	−0.002
	19	Breast	1454 HE Breast 50:50, Sun Nuclear	Tissue (soft)	1.002	0.987	0.001	−0.003	0.000	0.000
3	20	Polypropylene (PP)	TECAFINE PP natural, Ensinger	Non-tissue	1.003	0.972	−0.040	−0.007	0.010	−0.004
	21	Polymetylpentene (PMP)	TPX-RT18XB, Mitsui Chemicals, Minato, JP	Non-tissue	1.003	0.886	−0.078	0.000	0.013	0.004
	22	Lung 450	485 Lung LN-450, Sun Nuclear	Tissue (soft)	1.000	0.481	0.001	0.007	0.001	0.011
	23	Lung 300	455 Lung LN-300, Sun Nuclear	Tissue (soft)	1.000	0.282	0.000	0.003	0.000	0.003
	24	Air (no insert)		Tissue (soft)	–	0.001	0.001	−0.003	−0.001	0.004

The materials are grouped by phantom section in descending *SPR_ref_* (positions 1–24) as arranged in the three sections of the phantom (see Fig. 1). The inserts were classified as non-tissue materials or tissue surrogates, with the latter subdivided into bone and soft tissues. The table also presents SPR difference, calculated as (*SPR – SPR_ref_*) for Hounsfield look-up table (HLUT), DirectSPR, MMSim, and MD-SPR workflows. *k_E_* = energy correction factor (not applied for MMSim images).

**Figure 3:**
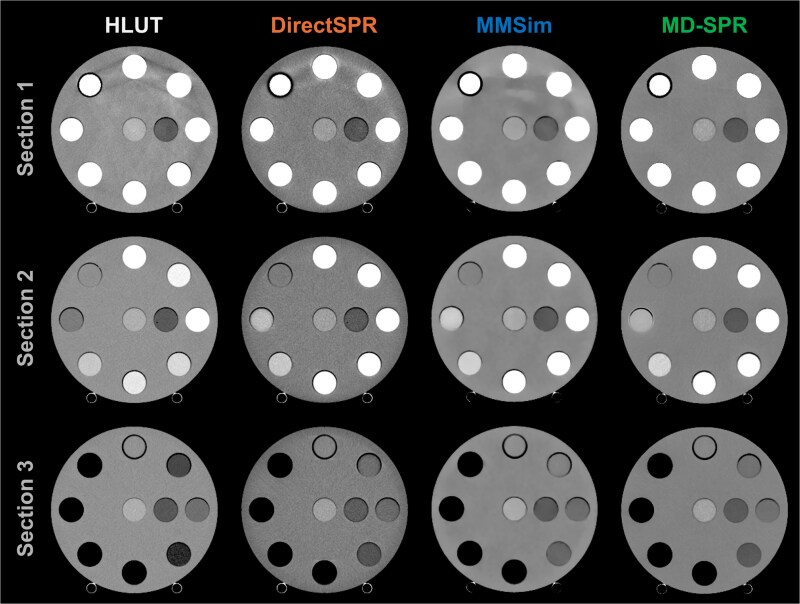
SPR images of the three sections in the 20 cm diameter phantom, created using HLUT, DirectSPR, MMSim, and MD-SPR workflows. The slice thicknesses varied slightly between the different CT techniques, as described in Table 1. The images are displayed with a greyscale window spanning 0.9–1.1 (unitless), selected to visualise artefacts between the inserts. Note that the dark ring around the bone cement insert (position 8 in Fig. 1) in Section 1 is surgical tape used to ensure a tight fit in the phantom.

The SPR images from HLUT, DirectSPR, and MMSim contained visible image artefacts in Section 1 of the phantom (Fig. 3). The artefacts appeared mainly as streaks between the three highest ${SPR}_{ref}$ inserts (PTFE, Cortical bone, and CB2 + 50% CaCO_3_) at positions 1, 2, and 3, and the bone cement at position 8 (see insert positions in Fig. 1). The direction and intensity (dark or bright) of the streaks differed between the different workflows.

Artefacts in the HLUT SPR images were already present in the original 120 kV SECT image (Fig. 2). For DirectSPR, the primary source of the artefacts could be traced back to the 80 kV image, whereas for MMSim both RED and EAN images contributed (Fig. 2). The water and iodine MD images from the FKS-DECT scans were relatively free of artefacts, producing MD-SPR images with minimal artefacts.

The ranges of the predicted SPR values for the HE General Adipose insert across the three sections of the phantom were 0.005, 0.005, 0.004, and 0.002 for HLUT, DirectSPR, MMSim, and MD-SPR, respectively. For HE Brain, the corresponding ranges were 0.006, 0.003, 0.005, and 0.001, respectively (Table 3).

**Table 3:** Predicted SPR values for the HE General Adipose and HE Brain inserts across the three phantom sections for each of the four SPR prediction workflows.

		CT predicted SPR		
Material	Workflow	Section 1	Section 2	Section 3
HE General Adipose	HLUT	0.972	0.969	0.968
	DirectSPR	0.967	0.970	0.972
	MMSim	0.975	0.972	0.971
	MD-SPR	0.968	0.970	0.970
HE Brain	HLUT	1.042	1.036	1.036
	DirectSPR	1.021	1.024	1.024
	MMSim	1.027	1.032	1.031
	MD-SPR	1.025	1.026	1.026

**Figure 4:**
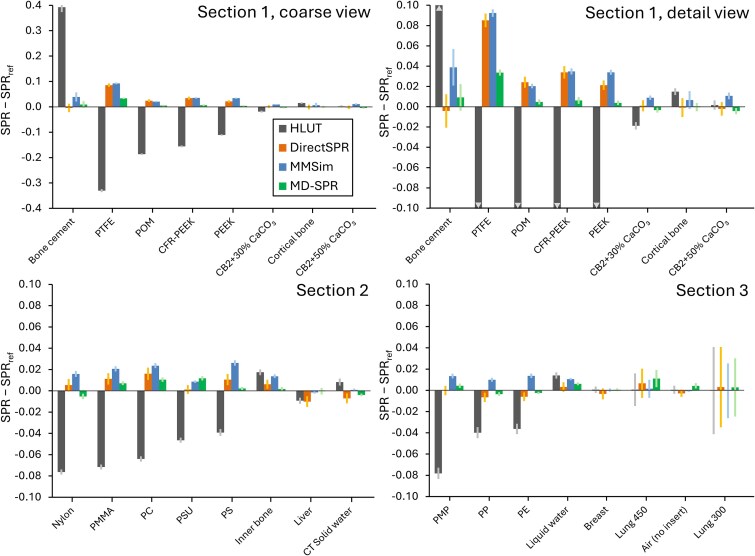
Difference between predicted and reference SPRs (i.e. SPR – SPR_ref_). Results are shown per phantom section, ordered by decreasing SPR difference for the HLUT workflow. Error bars represent ± the pooled standard deviation within the VOIs, across the three repeated scans. For abbreviations, see Table 2. Note that the HLUT values were truncated in the detail view of Section 1 (upper-right panel).

HLUT generally underestimated the SPRs for the non-tissue materials, except for bone cement (Table 2 and Fig. 4). The RMSEs for the non-tissue materials (listed in Table 2) using HLUT, DirectSPR, MMSim, and MD-SPR were 0.167, 0.028, 0.034, and 0.011, respectively. With HLUT, the largest SPR deviations for individual materials were observed for PTFE and bone cement at −0.330 and +0.393, respectively. With MD-SPR, the corresponding deviations for these materials were +0.034 and +0.009.

For all tissue surrogates (see included materials in Table 2), the RMSEs with HLUT, DirectSPR, MMSim, and MD-SPR were 0.011, 0.005, 0.007, and 0.005, respectively. For the four bone surrogates, the corresponding RMSEs were 0.015, 0.003, 0.010, and 0.003, whereas for the soft tissue surrogates the RMSEs were 0.007, 0.006, 0.004, and 0.005 (Table 2).

The largest SPR standard error of the mean for the three repeated scans using HLUT, DirectSPR, MMSim, and MD-SPR were 0.00012, 0.00026, 0.00074, and 0.00041, respectively.

## Discussion

This study evaluated four SPR prediction workflows based on different CT techniques, focusing on image artefacts and SPR accuracy using a commercial phantom with custom inserts.

SPR images from the MD-SPR workflow exhibited less pronounced streak artefacts compared with images from the other workflows, as observed both visually and through quantitative evaluation of the HE Brain and HE General Adipose inserts (placed near the centre of the phantom, outside the main artefacts). The absence of artefacts was not attributable to the MD-SPR model itself, but rather to the lack of artefacts in the underlying water and iodine MD images. For the SPR images from the HLUT, DirectSPR, and MMSim workflows, artefacts were particularly evident in Section 1 of the phantom, i.e. the section containing the materials with the highest SPRs. Artefacts in the HLUT SPR images were also visible in the 120 kV SECT images. For the DirectSPR images, the artefacts could be traced to the lower-energy 80 kV scan, consistent with AAPM TG-291 [13], which states that artefacts in DECT are strongly influenced by the accuracy of the lower energy scan data acquisition. The appearance of artefacts in the SPR images from the MMSim workflow was somewhat unexpected. DLCT, like the FKS-DECT technique, is expected to perform basis material decomposition in the projection-space, i.e., a technique that should reduce beam hardening artefacts. More pronounced artefacts were observed in the images based on the DLCT technique compared with those based on FKS-DECT, which suggests that the observed artefacts can be attributed to the methods used to generate the RED and EAN images from the DLCT scans. The implication for the resulting SPR images could be simplified to the observation that bright streaks in the RED images led to bright streaks in the SPR images, and conversely that dark streaks in the EAN images led to bright streaks in the SPR images. This indicates that the artefacts were generally not a result of the SPR prediction algorithms themselves but originated earlier in the imaging chain. To the best of our knowledge, this is the first published report of SPR images produced by MMSim.

All four workflows predicted SPRs within ±0.02 of ${SPR}_{ref}$ for the tissue surrogates, where the largest deviations were observed for HLUT in the bone surrogates. MD-SPR outperformed both DirectSPR and MMSim in terms of SPR accuracy for the non-tissue materials. This is likely attributable to the use of look-up tables to determine the RSN from the EAN, which is used by both DirectSPR and MMSim [15, 34] but avoided in the MD-SPR surface fitting approach. Two previous studies reported relative errors compared with measurements using a Peakfinder system for PEEK, POM, PMMA, and PTFE of 0.8, 1.3, 0.8, and 3.4% [21], and 1.7, 2.2, 1.3, and 4.4% [22], respectively. The corresponding relative errors for MD-SPR were 0.3, 0.4, 0.6, and 1.9%, respectively (recalculated to relative errors based on the data in Table 2). In this work, the SPR accuracy is expressed as the absolute SPR difference from ${SPR}_{ref}$, rather than relative SPR error, because this metric directly relates to the range of the proton beam in water. The SPR difference for a material, as reported in Table 2 and Fig. 4, represents the error in the water-equivalent range per centimetre of the path length through the material. For example, the water-equivalent range in 1 cm PTFE calculated using HLUT is 0.33 cm shorter than the range calculated using the reference SPR (see Table 2).

The improved SPR prediction accuracy achieved with the DECT-based workflows, compared with the SECT-based HLUT, enables smaller clinical range uncertainty margins. Such a reduction will, in turn, reduce dose to healthy tissues and thereby lower the risk of radiation-induced complications. A particularly challenging scenario involves bone cement, which can be difficult to distinguish from bone in SECT images. If an HLUT workflow is used without manual delineation and CT number override of the bone cement, a 1 cm layer of bone cement would cause a 0.4 cm overshoot in water-equivalent range compared with an uncorrected treatment plan. Such an overshoot may have clinically significant consequences for organs at risk located behind the implant. For non-tissue materials, the DECT-based workflows demonstrated an even greater improvement in accuracy than for the tissue surrogate materials, with the MD-SPR workflow yielding the highest overall accuracy. Accurate SPR prediction for materials such as polymer implants could remove barriers to proton therapy in patient selection and beam angle optimisation. Furthermore, improved SPR accuracy for non-tissue polymers opens new possibilities for material selection when designing phantoms used in research and quality control.

The MD-SPR model, originally presented by Pettersson *et al.* [25], was slightly revised to incorporate updated elemental I-values. In the previous work, both the MD-SPR model and theoretical SPR values used for evaluation were calculated using the same set of I-values, making the choice of I-values inconsequential when evaluating the precision of the model. In this study, the predicted SPRs were instead compared with measured ${SPR}_{ref}$. The main impact of using the ICRU-recommended I-values is slightly higher theoretical SPRs for bone tissues, due to the lower I-value for calcium in ICRU Report 49 [30] (216 eV) compared with Bär *et al.* [36] (258 eV).

The energy correction factor ${k}_E$ for establishing ${SPR}_{ref}$ was introduced to account for the energy dependence of the SPR when comparing the predicted SPRs with ${SPR}_{ref}$. The energy dependence has been reported to account for up to 0.4% relative uncertainty in SPR for bones [34]. Other studies, such as Möhler *et al.* [23] adjusted the energy of their SECT- and DECT-based SPR prediction workflows to the beam energy used for the measurements. In this study, since both the clinical HLUT and DirectSPR were set to 100 MeV, we adjusted the measured reference SPR instead.

The cylindrical phantom did not fully represent patient anatomy. Nevertheless, accurate depiction of all objects in an image is a prerequisite for accurate dose calculation in patients. The highest density materials were placed in the same section of the phantom to deliberately challenge the CT techniques under different beam hardening conditions, enabling comparison of artefacts in images of an inhomogeneous but well-centred cylindrical phantom. To further evaluate systematic biases in the SPR prediction accuracy from the different workflows, each material insert should also be examined individually under simplified beam hardening conditions, that is, in a homogeneous phantom of a low-EAN material. This should remove the dependence on the insert configuration and thus separate the impact of artefacts from the accuracy of the workflows.

Instead of using the original 16.5 cm long inserts of the commercial AED phantom, an additional set of tissue surrogate inserts was cut into 5 cm long pieces and used in this work. This enabled the use of the same individual inserts for both CT scans and proton beam measurements. Furthermore, materials of interest, such as bone cement, could be chosen more freely. However, the limited availability of bone cement restricted the insert size, and the images revealed small air cavities within the insert, which may have led to an underestimation of ${SPR}_{ref}$. Therefore, to ensure a fair comparison between the SPR prediction workflows, care was taken to ensure consistent VOIs across all SPR images.

None of the DECT-based workflows in this study required calibration scans. Vendor-provided DECT-based SPR prediction algorithms are expected to relieve individual clinics of the burden of calibrating their CT scanners [6]. However, SPR images should still be validated prior to their clinical introduction and subjected to periodic quality assurance. The MD-SPR model is based on the assumption that the water and iodine MD images are accurate. Therefore, the accuracy of these images should be evaluated before clinical implementation, particularly in anatomically complex regions such as the shoulders, which differ from cylindrical phantom geometries. At the time of writing, there is no commercial SPR algorithm available from GE Healthcare, although several potential methods have been published [18, 25, 41, 42].

Future research should refine SPR prediction by addressing several key areas. In addition to scanning the material inserts individually in simplified geometries, as previously mentioned, further evaluations under extreme worst-case conditions should be performed. A comparative analysis of tissue surrogates from different electron density phantoms used in the literature would also be valuable for understanding material-dependent discrepancies. In addition, the preservation of geometric features in SPR images, as well as the influence of iodine-based contrast materials, is of interest for future investigations.

## Conclusion

This study demonstrates how three DECT-based SPR prediction workflows, using different DECT techniques and SPR prediction algorithms, can reduce image artefacts and improve SPR prediction accuracy compared with a conventional SECT-based HLUT workflow. Amongst these, MD-SPR yielded the least pronounced artefacts and the highest overall agreement with measured SPRs.

## Data Availability

Data supporting the findings of this study are available from the corresponding author upon reasonable request.
